# 

**Published:** 1891-03

**Authors:** 


### Communications.

### MISSOURI DENTAL COLLEGE.

*Editor of the American Journal of Dental Science:*

The twenty-fith annual commencement took place at Memorial Hall, St. Louis, Mo., March 12, 1891.

Prof. H. H. Mudd, Dean, addressed the graduates as follows:

*Gentlemen, graduates of the Missouri Dental College:*—You have much reason to be proud of your department of medicine. Special training has brought it to its present high position. So great has been the appreciation of this skill that it alone has commanded respect and deference. It is one of the best of the results of the modern idea in educational pursuits. The limitation of educational effort to lines of practical application. So successful has been this special training that the dental profession seemed for a time inclined to accept as a practical fact that the narrow limits put upon a man by such training was an advantage. The liberal culture of the more eminent men in your department, however, recognized the great danger of modern educational methods in perfecting certain faculties at the expense of the general development of the individual. They saw that the dentist should not be merely a mechanic, but that he must be a man something more than a skilled workman. The individual should predominate over his profession. The man must be greater than his work, and the gentlemen who organized the Missouri Dental College fortunately appreciated the value of anatomy, chemistry and physiology in the education of a dentist. They recognized the danger of the modern tendency to specialism in the education of the individual. They knew that if a man was fitted into a groove, no matter how perfectly he filled the position, that his horizon was narrowed, and that he become dwarfed in body and limited in mental activity. A liberal education broadens the view, multiplies the avenues of growth, and helps to perfect the individual. Individual greatness exalts all mankind. Fortunately we have grand models—Divine and human—as examples. Strive for perfection, for your individual responsility; you are part of a stupendous whole, an atom among millions. You attract and repel, you influence and are influenced by others, and your life belongs not only to you and to your family, but to the world and the age. Be careful, then, lest now, in the period of activity, you do violence to the better sentiments of your youth and embitter the memories of the later years of life; for the record of time is surely written in your heart. The gaint tree of the forest has inner circles of growth that mark the rolling years. So you will find, as advancing years dull your perceptions and whiten your locks, that your play and your work have fashioned that inner life into harmony with your daily thoughts and actions. Eminence, then, in special work should not entail loss of power in the individual, but should be the outcome of a liberal education combined with a special effort in a given direction.

You have the honor to enter the profession from a school which was the first one of the United States to recognize in a practical way the importance of the thorough education to be obtained by a union with an established medical college. With singular unanimity and in advance of the general profession; the department of dentistry has reached a high and liberal basis for educational qualifications, and now demands of all dental colleges an obligatory three years' course. This demand will be enforced throughout the country after the close of the present session.

I congratulate you upon your fellowship in medicine in the special department of dentistry. Accept this token, then, as evidence of your right and of the belief of the faculty of the Missouri Dental College in your capacity to work in the field of dentistry.

The degree of Doctor of Dental Surgery was conferred upon the following named gentlemen:

Edward L. Beatie, Higginsville, Mo.; Canute L. Brudewald, Beaver Falls, Penn.; Arthur C. Bedford, Bloomfield, Mo.; M. C. Boswell, Wright City, Mo.; James S. Coyle, St. Louis, Mo.; Marvin L. Cumming, Farmer City, Ills.; Charles E. Dungan, Springfield, Ills.; W. R. Eckle, Lexington, Mo.; James B. Harrison, New Albany, Ind.; George D. Kennedy, Colorado Springs, Colo.; James B. Newby, St. Louis, Mo.; John W. Markwell, Coryden, Ind.; John E. Masterson, Madisonville, Texas; Ruben G. Porter, Petoski, Mich.; Jasper DeG. Peak, Osage City, Kan.; A. S. Purdy, Carbondale, Ills.; William T. Rutledge, Monroe City, Mo.; George C. Schwarz, Edwardsville, Ills.; Samuel Schrantz, Warrenton, Mo.; Carl E. Schumacher, St. Louis, Mo.; Otto Settellen, Basle, Swltzerland; Herman Saxermeyer, Red Bud, Ills.; John H. Thiele, Hanover, Germany; H. G. Voorhies, Moberly, Mo.; J. Warren Wick, St. Louis, Mo.; James F. Wallace, Argola, Mo.; Matthew D. Wilson, Lexington, Mo.; P. H. Winans, Pittsfield, Ills.

Matriculates, 90.

Dr. W. N. Morrison, President of the St. Louis Dental Society, awarded the prizes.

"The St. Louis Dental Society Prize."—An elegant gold medal, to William T. Rutledge, D. D. S., receiving the highest vote on final examination.

"The J. W. Wick Prize."—Twenty-five dollars in gold, to George D. Kennedy, D. D. S., receiving next to the highest vote on final examination.

"The S. S. White Dental Manufacturing Company Prize."—A set of Varney Pluggers, to Ruben G. Porter, D. D. S., excelling in Operative Dentistry.

"St. Louis Dental Manufacturing Company Prize."—A Laboratory Lathe, to James F. Wallace, D. D. S., for the best specimen case of Artificial Teeth.

---

### MISSOURI DENTAL COLLEGE ALUMNI.

The annual meeting of the Alumni Association of the Missouri Dental College was held March 12, 1891, at the College building, corner Seventh street and Clark avenue. Ten new members were elected to membership, including Drs. Newby and Wick, of this city, the others being members of the class of '91.

Officers for 1891 were elected as follows: President, Dr. W. M. Bartlett; Vice-President, Dr. J. H. Prothero; Secretaey, Dr. De Courcy Lindsley; Treasurer, Dr. Carl E. Schumacher; Executive Committee, Drs. Bowman, Prosser and Whipple.

During the past year the following alumni have been lost by death: Drs. Homer Judd, M. D. La Croix, John W. Forden and Henry D. Field. The following committee were appointed to draft suitable resolutions regarding the foregoing, to be presented at the next annual meeting: Drs. W. H. Eames, A. H. Fuller and John G. Harper. The by-laws were changed, doing away with annual dues.

---

### ILLINOIS STATE DENTAL SOCIETY.

*Editor of the American Journal of Dental Science:*

The following program is arranged for the Illinois State Dental Society, to be held at Bloomington, May 12, 13, 14 and 15, 1891.

I. Annual Address by the President, Dr. Truman W. Brophy, of Chicago.

II. Report of Committee on Dental Science and Literature by Dr. J. D. Moody, of Mendota, chairman.

III. Report of Committee on Dental Art and Mechanism by Dr. W. B. Ames, of Chicago, Chairman.

IV. How to Deal with the Condemned Pulp, by Dr. J. G. Dickson, of Carme. Discussion to be opened by Dr. J. Y. Reid, of Chicago.

V. The Preparation of Teeth for Filling by Dr. Edmund Noyes, of Chicago. Discussion to be opened by Dr, George H. Cushing, of Chicago.

VI. Prosthetic Dentistry by Dr. W. T. Magill of Rock Island. Discussion to be opened by Dr. E. C. Stone, of Galesbury.

VII. Third Period in the History of Dentistry, continued with biographical notes, by Dr. John J. R. Patrick, of Belleville.

VIII. Experimental Studies on the Action of Diffusible Medicinal Agents in Living and Pulpless Teeth, by

Dr. A. W. Harlan, of Chicago. Discussion to be opened by Dr. P. J. Kester, of Chicago.

IX. Architecture of the First Upper Molar, by Dr. Alton H. Thompson, of Topeka, Kansas. Discussion to be opened by Dr. A. B. Fruman, of Chicago.

X. A Lantern View of the Pulp Chambers and Canals, showing Typical Forms and Variations, by Dr. D. M. Cattell, of Chicago. Discussion to be opened by Dr. G, V. Black, of Jacksonville.

XI. Efficiency and Simplicity in Regulating Appliances, by Dr. E. H. Angle, of Minneapolis, Minn., illustrated by stereoptican. Discussion to be opened by Dr. C. S. Case, Jackson, Mich.

XII. Low Fusing Continuous Gum Body, by Dr. George Cunningham, of Cambridge, England. Discussion to be opened by Dr. A. B. Ames, Chicago.

XIII. Report of Supervisor of Clinics, by Dr. Louis Ottofy, of Chicago, chairman; with discussion.

CLINICS.

Dr. W. O. Kulp, Davenport, Iowa, Art Technique; Dr. J. P. Wilson, Burlington, Iowa, Root Filling; Dr. R. H. Mace, Belleville, Ill., Gold Crown; Dr. W. H. Taggert, Freeport, Ill., Filling at the Cervical Border, using extension arm cervix clamp; also a new Suspension Removable Bridge.

Dr. W. B. Ames will demonstrate Dr. Cunningham's Low Fusing Body for Continuous Gum.

Dr. Edgar Palmer, La Crosse, Wis., Administration of Narcotic Vapors, using valved inhalers.

Dr. A. O. Hunt, Iowa City, Iowa; a New Bridge; Dr. E. E. Hughes, Des Moines, Iowa, Gold Inlay; Dr. George H. Slyfield, Waukyhn, Ill., Contour Gold Filling.

Dr. K. B. Davis, Springfield, Ill., Artificial Crown; Dr. S. F. Duncan, Joliet, Ill., Method of Striking up Crown Tips.

It is particularly requested that those having pathological specimens, peculiar cases, models, new appliances and methods will bring them to the meeting.

All practitioners of Illinois (including non-members) and of other States are cordially invited to attend. They are especially urged to be present at the opening day and to remain through to the last session.

The usual reduction in hotel rates and railroad fares will be allowed.

J. W. WASSALL,
*Chairman Executive Committee.*

### To Readers and Correspondents.

Communications intended for publication, and exchange journals and papers, should be addressed to the Editor of The American Journal of Dental Science, No. 845 N. Eutaw St. Baltimore, Md. All letters relating to the business of the Journal, or containing cash remittances, to be directed to Messrs. Snowden & Cowman, No. 9 W. Fayette Street, Baltimore, Md. Articles for publication should be received by the Editor at least two weeks previous to the first of the month on which the number in which it is designed for them to appear, is issued.

☞ The American Journal of Dental Science will contain fifty pages of reading matter in each number, making a volume of about Six Hundred pages,

EXCLUSIVE OF ADVERTISEMENTS.

